# Childhood Stunting and Mortality Between 36 and 64 Years: The British 1946 Birth Cohort Study

**DOI:** 10.1210/jc.2012-3595

**Published:** 2013-03-26

**Authors:** Ken K. Ong, Rebecca Hardy, Imran Shah, Diana Kuh

**Affiliations:** Medical Research Council Unit for Lifelong Health and Ageing (K.K.O., R.H., I.S., D.K.), London WC1B 5JU, United Kingdom; and Medical Research Council Epidemiology Unit (K.K.O.), Institute of Metabolic Science, Addenbrooke's Hospital, Cambridge, CB2 0QQ, United Kingdom

## Abstract

**Background:**

Our aim was to examine the associations between childhood or adult height and adult mortality.

**Methods:**

In the prospective British 1946 Birth Cohort Study, childhood height was measured at 2, 4, 6, 7, 11, and 15 years, and adult height was measured at 36 years. Deaths were reported from the national health service register.

**Results:**

A total of 3877 study members (1963 male) contributed 106,333 person-years of follow-up; 391 deaths (228 male) were reported between the ages of 36 and 64 years. The strongest sex-adjusted association between height and mortality between ages 36 and 64 years was seen for height at age 6 years. The association was nonlinear; only study members in the shortest quintile at 6 years had a higher relative risk of adult mortality compared with those in the tallest quintile. By contemporary growth standards, 5.7% (n = 188) had heights at 6 years less than the second percentile, and a further 15.0% (n = 490) had heights between the second to ninth percentiles; these groups had higher adult mortality than all other study members (hazard ratio, 2.18; 95% confidence interval, 1.52–3.13; *P* < .001; and hazard ratio, 1.42; 95% confidence interval, 1.08–1.88; *P* = .01, respectively). Several determinants of childhood stunting (height at 6 years less than the second percentile) were directly associated with adult mortality; these included shorter parental heights and adverse early life nutrition and housing.

**Conclusions:**

British men and women born in 1946 were relatively stunted as children by contemporary standards. Those who were short at age 6 years had substantially higher mortality 30 to 60 years later. Furthermore, they accounted for the well-recognized inverse association between adult height and mortality.

Linear growth retardation (“stunting”) during early childhood is a marker of chronic undernutrition caused by poor nutrition and compounded by infectious disease (1). In deprived settings, stunting is associated with poor childhood outcomes, permanent deficits in adult height, poor adult health, and other adverse adult outcomes (1, 2). Furthermore, even across a wide range of settings, shorter adult height is consistently associated with higher risks of cardiovascular disease (CVD) and all-cause mortality in men and women (3–7). Understanding the association of poor childhood growth with mortality in contemporary aging populations is key to the developmental origins of disease hypothesis (8) and adds an important perspective to studies in contemporary birth cohorts, who are increasingly prone to rapid childhood weight gain and obesity (9, 10).

We hypothesized that short height in early childhood would be adversely associated with adult survival even in a high-income setting, that it would explain the association between shorter adult height and mortality, and that it would be independent of the association of weight status with mortality. We compared the associations between childhood or adult height and adult mortality in the British 1946 Birth Cohort Study. We also investigated whether adverse early childhood environments, poor nutrition, and physical illness, which are risk factors for poor childhood growth (11), might be related to adult mortality. Finally, we investigated whether adult risk factors might confound or mediate the relationship between height and mortality.

## Materials and Methods

### Study design

The Medical Research Council's National Survey of Health and Development (NSHD) is a socially stratified birth cohort of 2547 men and 2815 women of white European descent born during 1 week in 1946 who have been followed with repeated data collections since then (12). The study received Multi-Centre Research Ethics Committee approval, and informed consent was given by cohort participants.

Study members were flagged for death on the National Health Service Central Register from 1972. At that time, 2325 men and 2136 women were alive and residing in Britain; of the remaining cohort, 288 had already died, 586 had emigrated, and 27 were not flagged on the register. The underlying cause of death was coded according to the International Classification of Diseases (ICD)-9 or ICD-10 and were categorized as follows: cardiovascular diseases (ICD-9 codes 401–454 and ICD-10 codes I10–I89); cancers (ICD-9 codes 140–239 and ICD-10 codes C00–C97); externalizing disorders (including violent accidental and suicidal deaths; ICD-9 codes 291–292, 295–305, 307–309, 311–316, 570–571.3, 800–994, 1800–1869, and 1880–1999 and ICD-10 codes F61–F69, K70–K71, S00–X99, and Y85–Y98) and other causes. Cause of death was missing in 10 cases.

### Anthropometry

Birth weight to the nearest quarter pound (113 g) was extracted from medical records within a few weeks of birth and was converted to kilograms. Heights and weights were measured by trained personnel using standard protocols at ages 2, 4, 6, 7, 11, 15, 36, 43, 53, and 60 to 64 years and were self-reported at ages 20 and 26 years. Adult height was taken as the measured height at age 36 years; for 604 study members with missing data, we used self-reported height at age 26 years. Body mass index (BMI) was calculated as weight in kilograms divided by height in meters squared. Measures of BMI, weight, and height were converted into Z-scores using internally generated growth charts, constructed using the LMS method (13). To relate childhood height to current growth references, we also calculated height Z-scores by comparison with the World Health Organization (WHO) 2007 growth reference and defined stunting as height less than the second percentile (<−2 Z-scores) (14).

Mothers were, when possible, measured at the examination of their cohort child at age 6 years, and they reported their husband's height. Midparental height was calculated as an indicator of genetic height potential.

### Indicators of early childhood environment, nutrition, and illness

Various early life factors, namely, female sex, shorter midparental height, lower birth weight, higher birth order, higher number of young siblings, lower childhood (father's) social class, lower father's education, lower mother's education, and higher household crowding, have been examined previously as being potential determinants of adult height in this study (11). We examined those and other available nutritional factors as potential determinants linking childhood height to adult mortality. Childhood socioeconomic position (SEP) was based on father's occupation at age 4 years; for 159 study members with missing data, we used father's occupation at age 11 or 15 years. Fathers' and mothers' education levels were classified as at least secondary level or primary level with or without further education or qualifications (11). Information on breastfeeding (classified as never or ever breastfed) and number of lower respiratory tract infections were obtained from mother's reports to health visitors when children were aged 2 years. Family size (study member plus surviving siblings at age 2 years) and number of young children (aged <5 years) in the house were also recorded at age 2 years. Parity was based on the reported number of live births. At age 4 years, health visitors were asked to assess the mother's general management and cleanliness of the child and home. These responses were stratified in 3 groups: best; intermediate; and worst. Although these responses are subjective, they discriminated very poor environments that other measures failed to distinguish (15). Household crowding was calculated as the total number of people divided by the number of bedrooms or living rooms at age 4 years (11). Chronic illness between ages 0 and 4 years was defined as an illness that necessitated a hospital admission for at least 28 days. Information on energy and fat intake at 4 years was derived from maternal reports of what the child had eaten in the last 24 hours (16).

### Potential adult mediators and confounders

Height in childhood and adulthood is strongly related to adult SEP, smoking status, and lung function, which are themselves strong risk factors for mortality (17). Adult SEP was based on the head of household's occupation at age 36 years and classified as professional/intermediate, skilled nonmanual, skilled manual, or partly skilled/unskilled; for 768 study members with missing data, we used head of household's occupation at age 26 years. Smoking status (current smoker, ex-smoker, or never smoker) was available at 36 years; for 376 study members with missing data, we used smoking status at age 31 years. Systolic and diastolic blood pressure (BP) (millimeters of mercury), resting pulse rate, and peak expiratory flow rate (PEFR) (liters per minute) at 36 years were measured by nurses at home visits. BP was measured using a Hawksley random zero sphygmomanometer. The second of 2 measures was used unless the second recording was missing or invalid; then the first recording was used. PEFR was measured using a mini-Wright peak flow meter and was taken as the mean of the last 3 of up to 5 recordings.

### Analyses

Cox regression models were used to investigate the relationships between childhood or adult height Z-score and adult mortality, adjusted for sex. Follow-up time was from the cohort members' 36th birthday in 1982 until the first of death, emigration, or the end of February 2011 (just before the cohort's 65 birthday). If death had not occurred, follow-up was treated as censored. The proportional hazards assumption was checked graphically.

To explore the shape of the association with mortality, height Z-score was also entered as a quadratic term, and, if this term was significant, mortality rates were calculated by quintiles of height Z-score. We explored the interactions between childhood height and adult body size (height and BMI) by adding these additional interaction terms to the Cox regression models. Additional analyses of the association between childhood stunting and mortality during specific age periods from childhood were performed by logistic regression, with adjustment for sex, SEP, and smoking.

We tested which indicators of the early environment, childhood nutrition, and physical illness were independently associated with childhood stunting and whether these factors were also associated with adult mortality. Finally, further models for adult height Z-score and adult mortality were adjusted separately for potential adult confounders or mediators, including adult SEP, smoking status, weight, arterial BP, resting pulse rate, and PEFR. All analyses were performed using SPSS (version 15.0).

## Results

Details of the 3877 study members (1963 male and 1914 female) with data on adult height (at age 36 or 26 years) who were flagged on the national death register are summarized in Table 1. Of these, 391 deaths (228 male and 163 female) were recorded between ages 36 and 64 years (total 106,333 person-years follow-up; mean ± SD, 27.4 ± 5.0 years). The overall mortality rate was 3.7 deaths per 1000 person-years and was higher in men than in women (hazard ratio [HR], 1.39; 95% confidence interval, [CI], 1.13–1.70; *P* = .001).

**Table 1.** Description of the Population by Sex

	Male	Female	*P* Value*
Adult height, cm	175.7 ± 6.6 (1963)	162.4 ± 6.1 (1914)	<.001
Mother's height, cm	161.2 ± 6.4 (1732)	161.2 ± 6.6 (1668)	.9
Father's height, cm	173.2 ± 8.0 (1963)	173.4 ± 8.0 (1914)	.6
Follow-up from age 36, y	27.3 ± 5.2 (1963)	27.6 ± 4.7 (1914)	.04
Adult social class			
I (professional)	225 (11.6)	173 (9.1)	<.001
II (intermediate)	597 (30.8)	544 (28.7)	
IIIa (skilled nonmanual)	207 (10.7)	322 (17.0)	
IIIb (skilled manual)	632 (32.6)	538 (28.3)	
IV (partly skilled)	229 (11.8)	269 (14.2)	
V (unskilled)	50 (2.6)	52 (2.7)	
Cigarette smoking			
Never smoker	413 (25.0)	558 (33.5)	<.001
Current smoker	569 (34.4)	558 (33.5)	
Ex-smoker	670 (40.6)	550 (33.0)	
Systolic BP, mm Hg	122.8 ± 15.4	114.7 ± 15.6	<.001
Diastolic BP, mm Hg	78.4 ± 13.0 (1634)	73.3 ± 12.4 (1646)	<.001
Resting pulse rate, beats/min	70.9 ± 10.3 (1626)	73.0 ± 9.6 (1646)	<.001
PEFR, liters/min	554 ± 79 (1621)	412 ± 68 (1638)	<.001

Summaries are based on available data at age 36 years; where data are missing additional data on height and social class at 26 years and smoking at 31 years were used. Data are means ± SD (n) or n (%).

a *t* test for continuous variables; χ^2^ test for categorical variables.

### Childhood or adult height and all-cause mortality

Heights at all ages from 4 years onward were inversely associated with all-cause mortality between ages 36 and 64 years, after adjustment for sex (Table 2). In linear models, the strongest associations with mortality were seen with height at age 6 years (HR per +1 SD, 0.80; 95% CI, 0.72–0.89; *P* < .001) and adult height (HR per +1 SD, 0.80; 95% CI, 0.73–0.89; *P* < .001). In a combined multivariable regression model, height at 6 years (HR per +1 SD, 0.85; 95% CI, 0.73–0.99; *P* < .05) but not adult height (HR per +1 SD, 0.91; 95% CI, 0.78–1.06; *P* = .2) was associated with all-cause mortality between ages 36 and 64 years.

**Table 2.** HRs for All-Cause Mortality Between 36 and 64 Years per +1 SD Increase in Childhood or Adult Height

Age at Measurement	HR	95% CI	Total	Deaths
2 y	0.96	0.86–1.07	3133	319
4 y	0.87	0.79–0.97	3410	345
6 y	0.80	0.72–0.89	3276	328
7 y	0.82	0.74–0.91	3354	336
11 y	0.84	0.75–0.94	3282	330
14 y	0.82	0.73–0.92	2991	298
Adult height	0.80	0.73–0.89	3877	391

Values are adjusted for sex.

**Figure 1.**
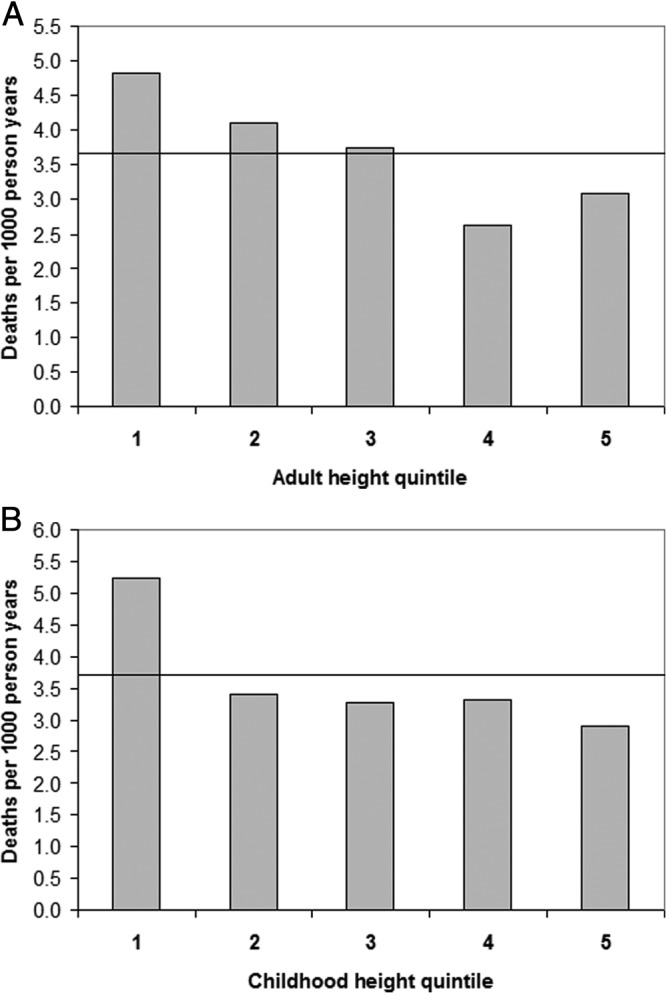
All-cause mortality during follow-up between 36 and 64 years by quintiles of adult height (A) and childhood height at age 6 years (B). The horizontal line indicates the overall mortality rate 3.7 deaths per 1000 person-years.

**Figure 2.**
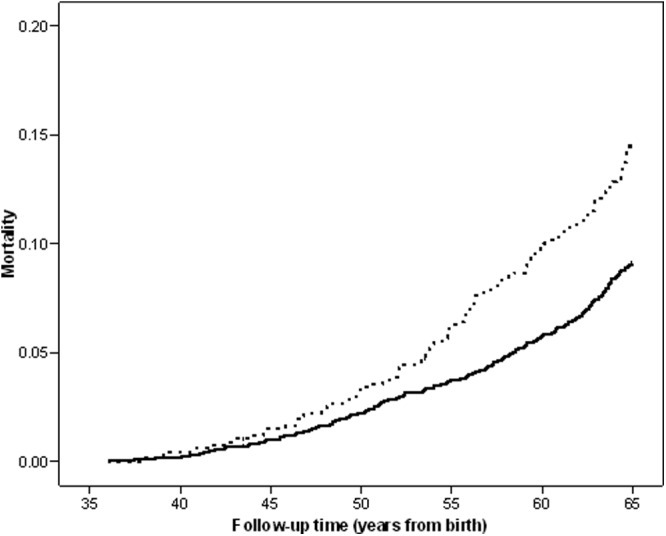
Kaplan-Maier plot of cumulative all-cause mortality between ages 36 and 64 years old in study members in the shortest height quintile at age 6 years (dotted line) vs those in any of the 4 taller height quintiles (solid line).

The shape of the associations between height at age 6 years or adult height and all-cause mortality were nonlinear, as indicated by the highly significant height-squared terms in the regression models (*P* < .001). Adult height showed a reverse J-shape association with the lowest mortality rate being in the second tallest quintile (Figure 1A). At age 6 years, higher mortality rates were only seen in the shortest quintile compared with the tallest (HR, 1.81; 95% CI, 1.30–2.54; *P* < .001) (Figures 1B and 2). Exclusion of those in the shortest quintile at 6 years nullified the association between adult height and mortality (HR per +1 SD, 0.94; 95% CI, 0.81–1.09; *P* = .4).

There was a significant interaction between height at 6 years and adult height with mortality (*P* = .01); taller adult height was protective against mortality only among those in the shortest height quintile at 6 years (HR per +1 SD, 0.72; 95% CI, 0.55–0.94; *P* = .01), but not among those in any of the taller childhood quintiles (HR per +1 SD, 0.94; 95% CI, 0.81–1.09; *P* = .4) (Supplemental Figure 1 published on The Endocrine Society's Journals Online web site at http://jcem.endojournals.org). There was weak evidence of interaction between short height at 6 years and adult BMI status (underweight, normal weight, overweight, or obese) and mortality (*P* for interaction = .08); the highest mortality rates were seen in those in the shortest height quintile at 6 years and subsequently either underweight (7.4 deaths per 1000 person-years; 15 deaths/n = 77 at baseline) or obese as adults (12.9 deaths per 1000 person-years; 14 deaths/n = 41 at baseline) (Supplemental Figure 2).

### Cause-specific mortality

Adult height was not associated with death from cancer, either in linear models (HR per +1 SD, 0.89; 95% CI, 0.77–1.04; *P* = .1; 171 deaths) or nonlinear models (not shown) but was inversely associated with deaths from CVD (HR per +1 SD, 0.78; 95% CI, 0.63–0.97; *P* = .02; 88 deaths), externalizing disorders (HR per +1 SD, 0.71; 95% CI, 0.54–0.94; *P* = .02; 50 deaths), and all other causes (HR per +1 SD, 0.72; 95% CI, 0.57–0.92; *P* = .007; 72 deaths). Similarly, being in the shortest vs all other quintiles at 6 years was not associated with a higher risk of death from cancer (HR per +1 SD, 0.72; 95% CI, 0.50–1.04; *P* = .08; 149 deaths), but showed similar sizes of associations with deaths from CVD (HR per +1 SD, 1.69; 95% CI, 1.05–2.74; *P* = .03; 79 deaths), externalizing disorders (HR per +1 SD, 1.90; 95% CI, 1.00–3.61; *P* = .05; 42 deaths), and all other causes (HR per +1 SD, 1.98; 95% CI, 1.14–3.44; *P* = .01; 56 deaths).

### Childhood stunting and its determinants associated with mortality

When height at 6 years was assessed against the contemporary WHO 2007 growth reference (14), all-cause mortality between 36 and 64 years was higher in those less than the second percentile, which is the height threshold currently used to define childhood stunting (6.8 deaths per 1000 person-years; HR, 2.18; 95% CI, 1.52–3.13; *P* < .001), and also in those between the second and ninth percentiles (4.7 deaths per 1000 person-years; HR, 1.42; 95% CI, 1.08–1.88; *P* = .01) compared with those with heights at 6 years that were taller than the ninth percentile (3.2 deaths per 1000 person-years) (Supplemental Figure 3). The association of stunting at age 6 years with earlier mortality (between ages 6 and 43 years: odds ratio (OR), 2.45; 95% CI, 0.92–6.55; *P* = .07; 105 deaths) was broadly similar to that with later mortality (between ages 43 and 63 years: OR, 1.69, 95% CI, 1.09–2.64; *P* = .02; 371 deaths).

Several indicators of the early environment, childhood nutrition, and physical illness were associated with risk of childhood stunting at age 6 years (Supplemental Table 1). Because these indicators were interrelated, we performed a forward stepwise regression model and identified 6 factors that were independently associated with being in the shortest height quintile at age 6 years. These factors were shorter midparental height, never breastfed, the presence of 3 or more young children (age <5 years) in the house, lower birth weight, a chronic illness requiring prolonged hospital admission before age 4 years, and intermediate or poor care of the house and child as assessed by the health visitor at age 4 years (Table 3).

**Table 3.** Independent Risk Factors for Childhood Stunting: ORs for Childhood Stunting and HRs for All-Cause Mortality Between 36 and 64 Years

	OR (95% CI) for Stunting^a^	HR (95% CI) for Adult Mortality^b^
Midparental height per SD	0.35 (0.27–0.46)	**0.85 (0.73–0.99)**
Breastfed: never vs ever)	1.75 (1.15–2.65)	**1.37 (1.08–1.73)**
3+ young children aged <5 y: yes vs no^c^	2.70 (1.55–4.71)	**1.53 (1.09–2.15)**
Birth weight per kg	0.58 (0.39–0.85)	0.95 (0.77–1.17)
Chronic illness <4 y: yes vs no^d^	2.59 (1.39–4.83)	1.16 (0.77–1.74)
Care of house and child^e^		
Best	Reference	Reference
Intermediate	1.44 (0.85–2.44)	1.13 (0.85–1.50)
Worst	1.79 (1.11–2.88)	1.04 (0.79–1.37)

a OR (95% CI) from the final multivariable model for childhood stunting, defined as height Z-score at age 6 years <−2 (equivalent to less than the second percentile) according to the WHO 2007 growth standard. Only those determinants that remained in the final model, with significant independent associations with stunting, are shown. The full list of potential determinants that were entered into the initial model is shown in Supplemental Table 1.

b HR (95% CI) for mortality between age 36 and 64 years, adjusted for sex, adult social class, and smoking. Significant (*P* < .05) associations are highlighted in bold.

c Number of young children (aged <5 years) in the house was recorded at age 2 years.

d Chronic illness was defined as an illness that necessitated a hospital admission for at least 28 days between ages 0 to 4 years.

e Based on assessments by health visitors at age 4 years.

Of these 6 determinants of stunting, midparental height (HR per +1 SD, 0.85; 95% CI, 0.73–0.99; *P* = .04), never breastfed (HR, 1.37; 95% CI, 1.08–1.73; *P* = .009), and 3 or more young children in the house (HR, 1.53; 95% CI, 1.09–2.15; *P* = .01) were also associated with all-cause mortality at age 36 to 64 years after adjustment for adult SEP and smoking (Table 3).

### Potential confounders and mediators

The association between adult height and all-cause mortality was similar in men (HR per +1 SD, 0.80; 95% CI, 0.70–0.92) and women separately (HR per +1 SD, 0.81; 95% CI, 0.69–0.94) and was only modestly attenuated when adjusted for potential confounders, adult SEP and smoking (Table 4). Of the possible mediators, the association between adult height and all-cause mortality showed no attenuation when adjusted for adult weight, systolic and diastolic BP, or resting pulse rate but was partially attenuated with adjustment for adult PEFR (Table 4).

**Table 4.** HRs for All-Cause Mortality Between 36 and 64 Years per +1 SD Increase in Adult Height: Basic Models and Models Adjusted for Potential Confounders or Mediators

Model	HR (95% CI)	Covariables in Models	Total	Deaths
A				
Basic model	0.80 (0.73–0.89)	Sex	3877	391
Men only	0.80 (0.70–0.92)		1963	228
Women only	0.81 (0.69–0.94)		1892	163
B				
Basic model	0.83 (0.75–0.92)	Sex	3838	381
Adjusted model	0.86 (0.78–0.96)	+ Adult social class		
C				
Basic model	0.80 (0.72–0.89)	Sex	3618	357
Adjusted model	0.82 (0.74–0.91)	+ Smoking		
D				
Basic model	0.79 (0.71–0.89)	Sex	2754	286
Adjusted model	0.80 (0.70–0.92)	+ Parental heights		
E				
Basic model	0.81 (0.72–0.91)	Sex	3262	315
Adjusted model	0.78 (0.69–0.88)	+ Weight at 36 y		
F				
Basic model	0.81 (0.72–0.90)	Sex	3280	321
Adjusted model	0.82 (0.73–0.91)	+ Diastolic and systolic BP at 36 y		
G				
Basic model	0.80 (0.72–0.90)	Sex	3272	319
Adjusted model	0.82 (0.73–0.92)	+ Resting pulse rate at 36 y		
H				
Basic model	0.84 (0.75–0.94)	Sex	3259	312
Adjusted model	0.89 (0.79–1.00)	+ PEFR at 36 y		

## Discussion

In keeping with several previous studies (5, 18–21), we found that taller adult height was robustly related to lower risk of all-cause mortality, and this association was not confounded by SEP or smoking. Uniquely, compared with previous studies, we were able to show that the association with adult mortality was strongest with height in early childhood. A recent study similarly reported that the inverse association between height and risk of adult coronary heart disease was strongest at age 7 years; however, they only had data on heights between ages 7 and 13 years (22). Notably, the associations between height and mortality in our study were nonlinear and appeared to be largely explained by short height at age 6 years (corresponding to height less than the ninth percentile on current growth charts). Others have also reported nonlinear associations; for example, in a large Norwegian cohort only adult height up to 165 cm was related to higher all-cause mortality (23), but they did not have information on childhood heights.

The birth cohort study design allowed us to identify early life factors that were related to both childhood short height and adult mortality. Midparental height is commonly interpreted as a marker of the child's genetic height potential. Most of the variation in growth after age 2 years is estimated to be genetically determined (24, 25). Alternatively, it is possible that the associations with parental heights reflected some influence of the parents' own childhood environment; however, the association with mortality was independent of SEP. Lack of breastfeeding in the 1940s would have increased the risk of gastrointestinal and respiratory infections, as is seen in contemporary birth cohorts in lower income settings (26). Furthermore, the quality and availability of infant formula milk would have been poor, being largely based on evaporated milk (27). In contrast, in contemporary Western birth cohorts, formula milk–fed infants show faster growth rates, presumably due to increased nutritional intakes in settings of low infection risk (28). Having many young children in the household was the third factor associated with both childhood short height and adult mortality. Whereas this again could reflect the higher risks of early life infection and poor nutrition, the markers of environmental adversity clustered together, and it may be inappropriate to single out any specific factor in a birth cohort whose members were relatively deprived and short as children by today's standards (11).

In the British 1946 Birth Cohort Study, 38% of children grew up in poor environmental circumstances, defined as a combination of overcrowding, mothers who had only primary school education, and fathers in manual occupations, and many such adverse early life factors were associated with shorter adult height (11). Environmental conditions improved substantially in later-born UK cohorts who have taller childhood heights and higher childhood and adult BMI (29) and in whom the health consequences of childhood overnutrition and rapid growth may be more pertinent to the developmental origins of disease than undernutrition (9, 10). Our findings may, therefore, have more relevance to current birth cohorts in settings in which childhood stunting remains common (30) and has adverse short-term and long-term consequences (2). NSHD study members experienced transition to the obesogenic environment during their third and fourth decades (29), and previous analyses identified U-shaped associations between childhood and adult BMI and mortality (31). The effects of height and BMI on adult mortality appeared to be additive and probably represent distinct mechanisms. In those who were short as children, the avoidance of later underweight and obesity may be particularly beneficial for survival in terms of absolute risk reduction. Our findings may therefore be relevant to settings that are undergoing transition from undernutrition to overnutrition, resulting in a mismatch between early and later life environments (32).

Our study has a number of limitations. Adult and childhood height, their determinants, such as breastfeeding, and mortality are strongly socially patterned. Although we performed statistical adjustments for SEP, it is possible that residual effects remained. SEP in childhood has a strong influence on adult mortality (33), through many mechanisms that span disease prevention, detection, and management. Tall height may improve confidence and self-efficacy and increase “life chances,” with regard to workplace and social capital. Height is also positively correlated with cognition, and, notably, in the NSHD greater height growth during early childhood between the ages 2 to 4 years was particularly associated with cognitive development (34). However, the association between cognitive ability and mortality appears to be largely explained by adult SEP (33). Strong social patterning and other clustering of adverse early life factors also limits our ability to fully distinguish between the many potential determinants of short childhood height that lead to subsequent mortality or even to speculate whether infectious, nutritional, or genetic causes are responsible.

Rather than adult height per se, it is likely that some other physiological mechanism related to childhood or adult height explains the link to later mortality. Similar to some previous studies (5), we found that lower respiratory function partially mediated the association between height and mortality. There were too few adult respiratory deaths (n = 20) to allow a robust separate analysis of this outcome as has been studied by others (35); however, our findings suggest potential links between childhood and adult respiratory function and deaths from nonrespiratory disease. Whereas shorter adult height is associated with all the major CVD risk factors, such as serum cholesterol, BP, and smoking (36, 37), such markers do not appear to explain the associations between height and CVD or all-cause mortality (5, 35, 38). Causal modeling using genetic variants related to adult height may give insights into the putative biological processes that link short height to later mortality. Finally, our findings have important relevance for the design of long-term surveillance studies of adult disease and mortality after recombinant growth hormone treatment for childhood short height (39, 40). Our results show that it is inappropriate to simply compare mortality in these patients with that in the general population.

In conclusion, in a long-running British birth cohort study, the well-recognized association between shorter adult height and higher all-cause mortality was explained by short height during early childhood, which in turn appeared to reflect both genetic factors and early life adversity.
